# Musculoskeletal Disorders and Their Impact on Job Performance Among School Teachers in Buraydah City

**DOI:** 10.7759/cureus.50584

**Published:** 2023-12-15

**Authors:** Seham Alharbi, Nahla J Alghafes, Yasmeen A Alfouzan, Raghad I Alhumaidan, Farah Alassaf, Abdullah Aldhuwyan, Tameem A Alhomaid

**Affiliations:** 1 College of Medicine, Unaizah College of Medicine and Medical Sciences, Qassim University, Qassim, SAU; 2 College of Medicine, Qassim University, Qassim, SAU; 3 Family Medicine, Saudi Ministry of Health (MOH), Al Qassim, SAU; 4 Family Medicine, Qassim Health Cluster, Buraydah, SAU

**Keywords:** saudi arabia, job performance, musculoskeletal pain, musculoskeletal disorders, high school teachers

## Abstract

Introduction: Musculoskeletal disorders (MSD) pose a significant challenge to the well-being and productivity of individuals and various occupational groups, including teachers. Among teachers, the prevalence of MSD has raised concerns globally, impacting their daily activities and overall quality of life. Buraidah and Saudi Arabia, like many other regions, face the implications of this issue. This study aimed to explore the prevalence and associated risk factors of MSD among teachers in Buraydah, providing valuable insights into the extent of the problem and potential areas for intervention.

Methodology: An analytic cross-sectional study was conducted for three months, from April 1 to June 30, 2023, using the Arabic version of the standardized Nordic Musculoskeletal Disorder Questionnaire. This study was conducted in all schools in Buraydah City, Saudi Arabia. The study population was all schoolteachers (including principals, vice principals, etc.) in Buraydah City. The study analyzed responses from 648 teachers and 139 school workers using statistical tests, including chi-square tests and logistic regression models.

Results: The results indicated a notable prevalence of MSD among teachers, with a significant association found between age, gender, and major depressive disorder (MDD) and MSD. The study reveals that females are at higher risk of MSD compared to males, emphasizing the need for gender-specific interventions. Moreover, the presence of MDD is identified as a significant contributor to MSD among teachers. However, certain demographic and lifestyle factors, such as marital status, level of school, smoking habits, and fixed rest times, do not show significant associations with MSD. Although age and years of experience are correlated, only age is found to significantly contribute to MSD. Regular exercise and BMI also do not emerge as significant contributors, although a lack of exercise shows a marginal impact.

Conclusion: This study's findings have implications for educational institutions and policymakers, highlighting the need for tailored interventions to address MSD among teachers. It underscores the importance of ergonomic interventions, gender-sensitive approaches, and mental health support.

## Introduction

The term "musculoskeletal disorders" (MSDs) refers to a group of periarticular conditions that affect the musculoskeletal system and primarily cause functional discomfort and everyday pain [1,2]. Musculoskeletal disorders (MSDs) are more prevalent among schoolteachers, who are required to spend long periods of time standing, sitting, and engaging in repetitive tasks, such as grading papers or typing on a computer [3].

These disorders can affect the teacher's physical health, causing pain, discomfort, and movement limitations, ultimately impacting their ability to perform their job effectively [4]. In addition to physical discomfort, MSDs can significantly impact a teacher's mental health and job performance. Teachers with MSDs may experience increased stress and anxiety, reduced job satisfaction, and difficulty meeting their job demands [5]. Moreover, MSDs can lead to absenteeism and decreased productivity, ultimately impacting student learning and achievement [1,6].

In Saudi Arabia, a study conducted in 2020 by Alqahtani et al. [7] surveyed 261 high school teachers in Saudi Arabia and found that 73% of the respondents reported experiencing MSDs in at least one body part, with the highest prevalence being in the lower back (47.5%), neck (42.5%), and shoulders (33.3%). The study also found that female teachers reported a higher prevalence of MSDs compared to male teachers. They reported decreased work efficiency (73.2%), increased absenteeism (69.5%), and decreased job satisfaction (68.2%) [7]. In another study done in Saudi Arabia, 79.2% reported experiencing MSDs in at least one body part, with the highest prevalence being in the neck (68.3%) and lower back (59.4%). This study reported decreased productivity (68.3%), increased absenteeism (57.4%), and decreased job satisfaction (46.5%) [8].

The high prevalence of MSDs among teachers in Saudi Arabia indicates a need for further studies identifying what teachers are struggling with so that ergonomic and other interventions can be identified to help reduce this burden among teachers.

To our knowledge, there are no studies exploring MSDs among schoolteachers in Buraydah City, and therefore, there are no data on the prevalence of MSDs among Buraydah City school teachers, their risk factors, or their impacts on performance. This study aimed to explore the prevalence and associated risk factors of MSD among teachers in Buraydah City, Saudi Arabia. The findings of this study could guide measures and strategies such as ergonomic interventions, physical activity programs, and education and awareness campaigns to prevent and manage MSDs among schoolteachers in Buraydah City and Saudi Arabia in general.

## Materials and methods

Study design and settings

An analytic cross-sectional study was conducted for three months, from April 1 to June 30, 2023, in all schools in Buraydah City, Saudi Arabia, targeting all school teachers and other school workers in Buraydah City.

Sample size

The minimum sample size (n) was calculated as follows: 

n=Z^2^xPxQ/D^2^

Where: 

n: Calculated sample size

Z: The z-score for a 95% confidence level = 1.96. 

P: 50%, assumed proportion of participants for maximum sample size calculation

Q: (1 - P) = 50%.

D: The margin of error = 0.05.

n = (1.96)^2^x 0.5x0.5/(0.05)^2^ = 384

The minimum calculated sample size to achieve a precision of ±5% with a 95% confidence interval was 384 teachers. To compensate for possible inaccurate responses and the erroneous completeness of questionnaires, we recruited 400 teachers.

Sampling technique

A multistage random sampling technique was used to select participants. All schools in Buraydah city were divided into four clusters based on their location in the city (East, West, North, and South). Teachers from each cluster were further divided into two strata based on their gender (Male and Female). Finally, 50 teachers were selected by systematic random sampling from each subcluster, making 400 participants in total.

Data collection instrument and procedure

We used a validated, self-administered questionnaire with four parts. The first part of the questionnaire had questions regarding socio-demographics, such as age, gender, marital status, duration of experience in teaching, reported weight and height, medications or physical therapy for MSD, and any other diseases. The second part inquired about MSDs using the Arabic version of the standardized Nordic Musculoskeletal Disorder Questionnaire [9,10]. The third part inquired about the working conditions, how many standing hours per day, how many lectures, and what postures they took when teaching. The fourth part inquired about the effect of MSDs on daily life activities and work, absenteeism, and sick leaves.

The investigators visited schools, sought permission from authorities, and attended teachers’ morning staff meetings to recruit them. Before data collection, participants were given all information about the study, including the study aims and objectives, and invited to participate voluntarily.

Statistical analysis

Both descriptive and inferential statistical analyses of the data were carried out. Simple frequencies and percentages of the sociodemographic characteristics and other categorical variables were calculated and tabulated. Percentages were also calculated for multiple-answer questions. For continuous variables, median and IQR were reported as central tendency and dispersion measures, respectively. To find any significant association between categorical variables, Fischer’s exact test was applied and interpreted. For continuous variables, the Kruskal-Wallis test was used to compare medians. Furthermore, to predict factors causing MSD symptoms, a binary logistic regression model with multiple predictors was created. The results of the model were presented as adjusted odds ratios (AOR). Statistical significance was established at a p-value of 0.05 or less with a 95% confidence interval. All the statistical calculations were performed using IBM Corp. Released 2020. IBM SPSS Statistics for Windows, Version 27.0. Armonk, NY: IBM Corp.

Ethical considerations**

This study was approved by the ethics committee of Qassim province (Ref. No.: H-04-Q-001). Written consent was requested from participants before data collection. The study investigators requested approval from competent authorities and permission from the selected schools. There was no disclosure of the information obtained in this study to the hospital, legal or financial authorities, or anyone outside the study. The questionnaire collected anonymous information; no identifying data was collected, and participants had the right to withdraw. There was no disclosure of the information obtained in this study to anyone else outside of the study.

## Results

As indicated in Table 1, the total study participants were 787; among them, 648 were teachers, and the remaining 139 were other people working in school. The median age was 43 years, and the median years of experience were 16. The gender distribution shows 65.1% females and 34.9% males. Most participants (89.2%) were married. School level distribution was 29.9% high school, 22.2% intermediate, and 47.9% primary school. Non-smokers account for 94.4%. The BMI distribution includes 27.6% normal, 33.4% obese, 37.4% overweight, and 1.7% underweight participants. Regular exercise was undertaken by 41.7%, while 58.3% did not. Most (61%) had no chronic diseases, while 14.3% had osteoarthritis.

**Table 1:** Sociodemographic characteristics of study participants IQR: Interquartile range, N: Frequency, %: Percentage

		Total (N=787)		Teacher (N=648)		Other (N=139)	
		Median	IQR (Q_3_- Q_1_)	Median	IQR (Q_3_- Q_1_)	Median	IQR (Q_3_- Q_1_)
Age		43	8 (47-39)	43	7 (47-40)	43	5 (47-38)
Years of experience		16	11 (22-11)	17	10 (22-12)	14	14 (22-8)
		Count	Column N %	Count	Column N %	Count	Column N %
Gender	Female	512	65.1%	413	63.7%	99	71.2%
	Male	275	34.9%	235	36.3%	40	28.8%
Marital status	Divorced	30	3.8%	22	3.4%	8	5.8%
	Married	702	89.2%	588	90.7%	114	82.0%
	Single	45	5.7%	31	4.8%	14	10.1%
	Window	10	1.3%	7	1.1%	3	2.2%
Level of school you are working in	High school	235	29.9%	181	27.9%	54	38.8%
	Intermediate school	175	22.2%	153	23.6%	22	15.8%
	primary school	377	47.9%	314	48.5%	63	45.3%
Smoker	No	743	94.4%	614	94.8%	129	92.8%
	Yes	44	5.6%	34	5.2%	10	7.2%
BMI	Normal	217	27.6%	182	28.1%	35	25.2%
	Obese	263	33.4%	210	32.4%	53	38.1%
	Overweight	294	37.4%	248	38.3%	46	33.1%
	Underweight	13	1.7%	8	1.2%	5	3.6%
Regular exercise (at least 30min 5 time/week)	No	459	58.3%	385	59.4%	74	53.2%
	Yes	328	41.7%	263	40.6%	65	46.8%
Chronic disease	None	482	61%	394	60%	88	80.7%
	Hypertension	55	6.9%	47	8.4%	8	7.3%
	Asthma	41	5.2%	33	5.0%	8	5.5%
	Osteoarthritis	113	14.3%	102	15.7%	11	7.9%
	diabetes	81	10%	61	9.4%	20	14%
	Vertebral disc disease	12	1.5%	5	0.07%	7	5%
	Hypothyroidism	20	2.5%	16	2.5%	4	2.8%
	Other	28	3.5%	28	4.3%	0	0%

When asked about experiencing troubles such as aches, pains, discomfort, or numbness in the past 12 months, most (78.4%) of the other school staff and 83.3% of teachers responded positively. Among other school staff, 71.9% reported being prevented from carrying out normal activities due to these troubles, compared to teachers (73.6%). In the past 12 months, 55.4% of other staff and 59% of teachers had sought medical consultation, and within the last seven days, 83.5% of other staff and 79.9% of teachers reported still having the symptoms (Table 2). 

**Table 2:** Prevalence, impact, and severity of MSD in teachers and others working in school N: Frequency, %: Percentage

		Other (n=139)		Teacher (n=648)		p-value
		N	%	N	%	
In the past 12 months, have you had MSD problems (such as ache, pain, discomfort, numbness)	No	30	21.6%	108	16.7%	0.167
	Yes	109	78.4%	540	83.3%	
In the past 12 months, had you ‏been prevented from carrying out normal activities (e.g., job, housework, hobbies) because of this trouble	No	39	28.1%	171	26.4%	0.687
	Yes	100	71.9%	477	73.6%	
In the past 12 months, have you seen a physician for this condition	No	62	44.6%	266	41.0%	0.440
	Yes	77	55.4%	382	59.0%	
In the past seven days, did you suffer from any of the previous symptoms (other than sports injuries)	No	23	16.5%	130	20.1%	0.342
	Yes	116	83.5%	518	79.9%	
Treatment taken in last 12 months	None	25	18%	132	20%	0.567
	Rest	2	1.7%	8	1.5%	
	Salt and water	1	.5%	3	.5%	
	Message	41	29%	217	33%	
	Compresses	21	15%	109	16%	
	Physiotherapy	22	16%	117	18%	
	Oral analgesics	37	27%	196	30%	
	Topical analgesics	50	35%	264	40%	

**Figure 1:**
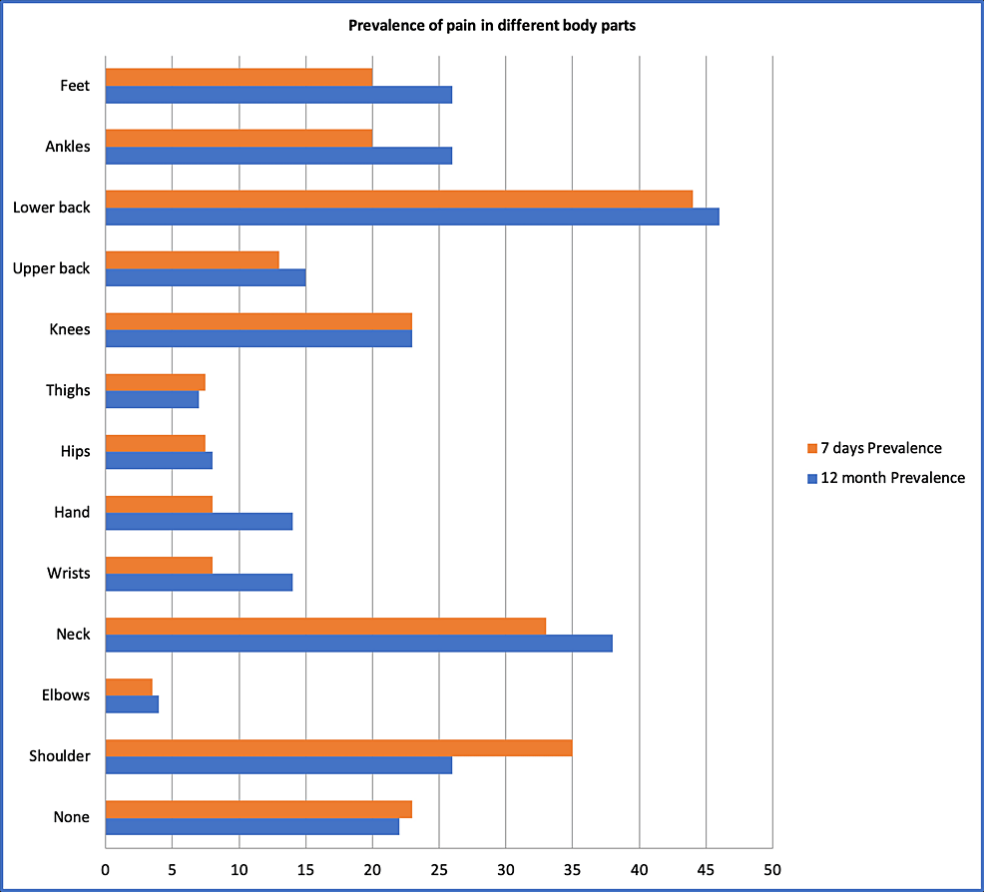
Prevalence of pain in different body parts

**Figure 2:**
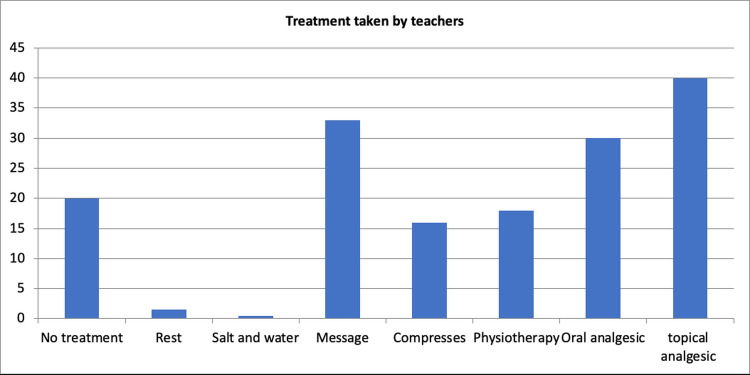
Treatment taken by teachers

The most prevalent musculoskeletal problem was lower back discomfort (46%), followed by neck pain (38%). Notably, shoulder, ankle, and foot troubles share a prevalence of 26% (Figure 1). Among the other school staff, those who experienced MSD in the past 12 months reported a median of two days absent from work due to muscle or joint pain, compared to 0 days for those who did not experience such pain. Among teachers, those who experienced MSD in the past 12 months reported a median of three days of absence, while those without such pain reported zero days. The most used treatments were topical analgesics (40%), massage (36%), and oral analgesics (30%) (Figure 2).

The teachers who considered changing their jobs showed a significantly higher percentage of pain compared to those who did not consider changing their jobs (p<0.001) (Table 3).

**Table 3:** Association of MSD with the performance of teachers and others working in school IQR: Interquartile range, N: Frequency, %: Percentage, MSD: Musculoskeletal disorder

				MSD in past 12 months				p-value
				No		Yes		
				Median N	IQR (Q_3_- Q_1_) %	Median N	IQR (Q_3_- Q_1_) %	
	Other	During the past 12 months, how many days have you been absent from work due to muscle or joint pain other than sports injuries?		0	0	2	5 (5-0)	<0.001
		Have you thought about changing your job due to muscle and joint pain?	No	28	93.3%	95	87.2%	.348
			Yes	2	6.7%	14	12.8%	
	Teacher	During the past 12 months, how many days have you been absent from work due to muscle or joint pain other than sports injuries?		0	1 (1-0)	0	3 (3-0)	<0.001
		Have you thought about changing your job due to muscle and joint pain?	No	100	92.6%	401	74.3%	<0.001
			Yes	8	7.4%	139	25.7%	

Table 4 shows that teachers who reported being prevented from normal activities, who reported aches, pain, discomfort, numbness, and due to MSD, and who sought physician consultation for MSD in the past 12 months were significantly more likely to suffer from major depressive disorders than those who did not report such problems (p<0.001, p<0.007, and p<0.018, respectively). Teachers with MSD symptoms within the last seven days were significantly more likely to have a major depressive disorder (p<0.001) (Table 4).

**Table 4:** Association of major depression with MSD among teachers N: Frequency, %: Percentage

		Major depressive disorder				p-value
		No		Yes		
		N	%	N	%	
In the past 12 months had you trouble (such as ache, pain, discomfort, numbness)	No	96	18.7%	12	9.0%	0.007
	Yes	418	81.3%	122	91.0%	
In the past 12 months have you ‏been prevented from carrying out normal activities (e.g., job, housework, hobbies) because of this trouble	No	155	30.2%	16	11.9%	0.001
	Yes	359	69.8%	118	88.1%	
In the past 12 months have you seen a physician for this condition of MSD	No	223	43.4%	43	32.1%	0.018
	Yes	291	56.6%	91	67.9%	
In the past seven days did you suffer from any of the previous symptoms (other than sports injuries)	No	118	23.0%	12	9.0%	0.001
	Yes	396	77.0%	122	91.0%	
Severity of disease	No	241	46.9%	69	51.5%	0.342
	Yes	273	53.1%	65	48.5%	

The chi-square test showed that MSD prevalence significantly increases with age (p<0.001). Females had a higher prevalence of MSD (67.0%) compared to males (33.0%) (p<0.001). Working hours, including fixed rest times (sitting), significantly affect MSD prevalence (p = 0.002), and years of job experience were significantly associated with MSD prevalence (p=0.041) (Table 5).

**Table 5:** Association of sociodemographic factors with MSD among teachers IQR: Interquartile range, N: Frequency, %: Percentage, MSD: Musculoskeletal disorder

		Any MSD in past 12 months				p-value
		No		Yes		
		Count/Median	%/IQR	Count/Median	%/IQR	
Age		40	8 (45-37)	44	7 (47-40)	0.001
Gender	Female	51	47.2%	362	67.0%	0.001
	Male	57	52.8%	178	33.0%	
Marital status	Divorced	3	2.8%	19	3.5%	0.069
	Married	95	88.0%	493	91.3%	
	Single	10	9.3%	21	3.9%	
	Window	0	0.0%	7	1.3%	
Level of school you are working in	High school	28	25.9%	153	28.3%	0.859
	Intermediate school	27	25.0%	126	23.3%	
	Primary school	53	49.1%	261	48.3%	
Years of job experience		15	11(21-10)	17	10(22-12)	0.041
Smoker	No	101	93.5%	513	95.0%	0.528
	Yes	7	6.5%	27	5.0%	
BMI	Normal	37	34.3%	145	26.9%	0.124
	Obese	27	25.0%	183	33.9%	
	Over weight	44	40.7%	204	37.8%	
	Under weight	0	0.0%	8	1.5%	
Regular exercise? (at least 30 min. 5 time/week)	No	58	53.7%	327	60.6%	0.186
	Yes	50	46.3%	213	39.4%	
Do your working hours include fixed, frequent and regular rest times (sitting)?	No	41	38.0%	126	23.3%	0.002
	yes	67	62.0%	414	76.7%	

A multiple regression model was created to evaluate the risk factors associated with MSD (Table 6). Age showed a significant association with MSD (aOR: 1.070, 95%CI: 1.009-1.136, p=0.025); each increase in age is associated with a 7% increase in the odds of experiencing pain. Females experience higher MSD compared to males (aOR: 2.581, 95%CI: 1.617-4121, p < 0.001). 

**Table 6:** Risk factors for MSD among teachers AOR: Adjusted odds ratio, C.I.: Confidence interval, IQR: Interquartile range, N: Frequency, %: Percentage

		Pain in last seven days				p-value	AOR	95% C.I. for AOR	
		No		Yes				Lower	Upper
		Count/Median	%/ IQR	Count/Median	%/ IQR				
Age		40	45-35	44	47-40	0.025	1.070	1.009	1.136
Years of experience		14	21-8	17	22-12	0.958	1.001	0.951	1.054
Gender	Female	56	43.1%	357	68.9%	<0.001	2.581	1.617	4.121
	Male	74	56.9%	161	31.1%				
Marital status	divorced	4	3.1%	18	3.5%	0.652	1.761	0.151	20.562
	Married	112	86.2%	476	91.9%	0.489	2.163	0.244	19.209
	Single	13	10.0%	18	3.5%	0.811	1.333	0.126	14.085
	Window	1	0.8%	6	1.2%				
Level of school you are working in	High school	38	29.2%	143	27.6%	0.834	0.948	0.948	0.577
	Intermediate school	34	26.2%	119	23.0%	0.549	1.181	1.181	0.686
	Primary school	58	44.6%	256	49.4%				
Smoker	No	120	92.3%	494	95.4%	0.346	0.664	0.283	1.558
	yes	10	7.7%	24	4.6%				
BMI	Normal	41	31.5%	141	27.2%	0.535	1.613	0.033	3.116
	Obese	29	22.3%	181	34.9%	0.681	1.375	0.037	3.642
	Overweight	59	45.4%	189	36.5%	0.653	1.413	0.023	2.157
	Underweight	1	0.8%	7	1.4%				
Regular exercise? (at least 30min 5 time/week)	No	67	51.5%	318	61.4%	0.382	1.214	0.786	1.874
	Yes	63	48.5%	200	38.6%				
Do your working hours include fixed, frequent and regular rest times (sitting)?	No	35	26.9%	132	25.5%	0.392	1.239	0.786	1.874
	Yes	95	73.1%	386	74.5%				

Regarding impacts of MSD and its associated factors, females had 2.906 times higher odds of experiencing disability due to MSD compared to males (p < 0.001) (Table 7).

**Table 7:** Impact of MSD and its association with various factors AOR: Adjusted odds ratio, C.I.: Confidence interval, IQR: Interquartile range, N: Frequency, %: Percentage, MSD: Musculoskeletal disorder

		Disability due to MSD				p-value	AOR	95% C.I.	
		No		Yes				Lower	Upper
		N/Median	%/ IQR	N/Median	%/ IQR				
Age		40	(45-37)	44	48-40	1.110	1.048	1.175	1.110
Years of experience		15	(20-10)	17	22-12	0.084	0.957	0.911	1.006
Gender	female	76	44.4%	337	70.6%	<0.001	2.906	1.886	4.477
	male	95	55.6%	140	29.4%				
Marital status	divorced	7	4.1%	15	3.1%	0.457	0.266	0.008	8.712
	married	146	85.4%	442	92.7%	0.893	0.813	0.039	16.743
	single	18	10.5%	13	2.7%	0.613	0.437	0.018	10.800
	window	0	0.0%	7	1.5%				
Level of school you are working in	High school	49	28.7%	132	27.7%	0.735	0.924	0.584	1.462
	Intermediate school	45	26.3%	108	22.6%	0.574	1.156	.698	1.913
	primary school	77	45.0%	237	49.7%				
Smoker	no	161	94.2%	453	95.0%	0.035	0.399	0.170	0.935
	yes	10	5.8%	24	5.0%				
BMI	Normal	63	36.8%	119	24.9%	0.074	0.113	0.010	1.238
	Obese	44	25.7%	166	34.8%	0.137	0.161	0.014	1.788
	Overweight	63	36.8%	185	38.8%	0.130	0.157	0.014	1.722
	Underweight	1	0.6%	7	1.5%				
Regular exercise? (at least 30 min. 5 time/week)	no	93	54.4%	292	61.2%	0.743	1.070	0.713	1.606
	Yes	78	45.6%	185	38.8%				
Do your working hours include fixed, frequent, and regular rest times (sitting)?	no	49	28.7%	118	24.7%	0.828	0.952	0.610	1.486
	yes	122	71.3%	359	75.3%				
Major depressive disorder	N0	155	90.6%	359	75.3%	<0.001	0.316	0.175	0.571
	Yes	16	9.4%	118	24.7%				

## Discussion

The study's findings shed light on the significant prevalence of MSD among teachers and other school staff and would also help educational institutions and policymakers take measures to promote safe and healthy working conditions for teachers to prevent the development of MSDs and improve their work performance. The gender distribution displayed a majority of females (65.1%), highlighting the gender composition in the teaching profession and in teachers with MSD reported by other studies [1]. The prevalence of MSD, as indicated by the self-reported symptoms, such as pain and discomfort, varied across body regions. Neck and lower back pain were particularly common, affecting 38% and 46% of participants, respectively. These results are comparable to other Saudi Arabian studies from Abba (59.2%) [11], Dammam (63.8%) [6], and a national-based survey (66.9%) [12]. In contrast, a Japanese study showed a lower prevalence of back pain (20.6%) [13]. Notably, the prevalence of MSD was high across multiple regions, underscoring the need for comprehensive interventions.

Our findings showed that most participants in both groups experienced symptoms such as aches, pains, discomfort, or numbness in the past 12 months. Among the other school staff, 78.4% reported such symptoms, while the teachers had a slightly higher prevalence at 83.3%, similar to the findings of other studies [3,13,14]. Furthermore, the study examined whether these symptoms prevented participants from carrying out normal activities. It was observed that 73.6% of the teachers were affected, and 59.0% of the teachers had seen a physician for their condition in the past 12 months. These findings align with another study conducted in Cairo, Egypt [15]. 

The findings from this study underscore the substantial impact of musculoskeletal disorder (MSD) on both work performance and individuals' job considerations. Among teachers, the median number of absent days for those with MSD was three days, while those without such pain reported none. This significant difference (p<0.001) further substantiates the impact of MSD on work attendance. A similar study conducted in Italy found that there was a greater intention to call off work and leave the job among those diagnosed with MSD [16], aligning with another study conducted in Qassam, Saudi Arabia, that also showed a significant relationship between absenteeism and pain [17]. Moreover, the association between job considerations and MSD among teachers was also significant (p<0.001). A higher percentage (94.3%) of participants contemplating job changes reported experiencing pain, highlighting the strong link between MSD and the inclination to explore alternative job options. Similar findings were reported by previous studies [13,18]. Altogether, these findings emphasize the multifaceted influence of MSD, encompassing both absenteeism and job-related decision-making, underscoring the importance of holistic interventions to mitigate its effects and enhance overall workplace well-being. Age exhibits a positive and significant association with MSD (p=0.025). This finding underscores the influence of age on the likelihood of experiencing pain and emphasizes the need for age-sensitive interventions. Similar findings were reported by other studies conducted in Saudi Arabia [6,18]. However, another study conducted in Saudi Arabia presents a contrasting result, showing no association of pain with age [19]. Gender emerges as a significant predictor, with females reporting higher MSD rates compared to their male counterparts. The substantial difference highlights the gender-based disparities in MSD experiences. These findings are comparable to the findings of other studies conducted in Saudi Arabia and Cairo [15,18]. Targeted interventions could enhance pain management strategies. Marital status, level of school, smoking habits, BMI categories, and fixed rest times were not significantly associated with MSD. These results suggest that these factors might not be associated with MSD. However, some of our study's findings contradict the previously conducted studies, which show that weight is significantly associated with MSD [6,17]. While BMI categories do not independently predict MSD, the lack of regular exercise marginally increases the odds of experiencing pain. Although not statistically significant, further studies might focus on this as a potential intervention. Interestingly, the presence of major depressive disorder significantly correlates with a higher MSD prevalence (P<0.001). This association signifies the intricate interplay between mental and physical well-being, emphasizing the need for holistic approaches to address both conditions simultaneously. Teachers with major depressive disorders reported more MSD-related symptoms, activity limitations, and medical consultations. This is in contrast to the results of a study that shows no correlation between depression and MSD [20]. However, MSD severity's association with MDD was not significant, underscoring the complex interplay between mental and physical health in educators. Age and years of experience, though showing a slight trend, do not exhibit a significant correlation with disability, aligning with another previous study indicating that these factors might not strongly influence such outcomes [17]. Gender emerges as a significant determinant, with females facing higher disability rates. This could be attributed to biological differences, differing job roles, or varied coping mechanisms. Participants with major depressive disorders exhibited significantly higher odds of experiencing disability due to MSD, emphasizing the need for comprehensive health assessments and integrated care approaches. Similar findings were also reported by the study, showing the negative impact of MSD on quality of life among elementary school teachers [21].

Some limitations of this study include its cross-sectional design, which is unable to establish causal relationships between risk factors and MSD. The reliance on self-reported data for MSD might introduce recall bias or social desirability bias, affecting the accuracy of reported pain levels and associated factors. Finally, the study's findings might not be easily generalizable to teachers in other regions or countries due to cultural, organizational, or educational system differences.

## Conclusions

This study showed a high prevalence of MSD among teachers, highlighting the importance of addressing this issue for the well-being of educators. The study identifies key risk factors associated with MSD, including age and gender. MDD was also found to influence MSD. These findings emphasize the need for targeted interventions to alleviate pain and promote the overall health of teachers. Considering the high prevalence of MSD among teachers, implementing ergonomic interventions is crucial. Designing classrooms with adjustable furniture and promoting proper posture during teaching could reduce the strain on muscles and joints. Given the association between age and MSD, interventions should be tailored to different age groups. Younger teachers might benefit from preventive measures, while older teachers could benefit from pain management strategies. Recognizing the gender-based differences in MSD, design interventions that address the unique needs of male and female teachers. This might involve providing targeted exercises, workshops, or resources. Since major depressive disorder is linked to higher MSD, adopting an integrated approach to mental and physical health is essential. Collaboration between healthcare professionals specializing in both domains can yield comprehensive solutions.
